# Practical considerations for choosing a mouse model of Alzheimer’s disease

**DOI:** 10.1186/s13024-017-0231-7

**Published:** 2017-12-22

**Authors:** Joanna L. Jankowsky, Hui Zheng

**Affiliations:** 1 Department of Neuroscience, Baylor College of Medicine, Houston, TX 77030 USA; 2 Department of Neurology, Baylor College of Medicine, Houston, TX 77030 USA; 3 Department of Neurosurgery, Baylor College of Medicine, Houston, TX 77030 USA; 4 Department of Molecular and Cellular Biology, Baylor College of Medicine, Houston, TX 77030 USA; 5 Huffington Center on Aging, Baylor College of Medicine, Houston, TX 77030 USA; 6 Department of Molecular and Human Genetics, Baylor College of Medicine, Houston, TX 77030 USA

**Keywords:** Transgenic mouse, Knockout, Knock-in, Amyloid precursor protein, APP, Aβ, Tau, MAPT, TREM2, Apolipoprotein E, ApoE

## Abstract

Alzheimer’s disease (AD) is behaviorally identified by progressive memory impairment and pathologically characterized by the triad of β-amyloid plaques, neurofibrillary tangles, and neurodegeneration. Genetic mutations and risk factors have been identified that are either causal or modify the disease progression. These genetic and pathological features serve as basis for the creation and validation of mouse models of AD. Efforts made in the past quarter-century have produced over 100 genetically engineered mouse lines that recapitulate some aspects of AD clinicopathology. These models have been valuable resources for understanding genetic interactions that contribute to disease and cellular reactions that are engaged in response. Here we focus on mouse models that have been widely used stalwarts of the field or that are recently developed bellwethers of the future. Rather than providing a summary of each model, we endeavor to compare and contrast the genetic approaches employed and to discuss their respective advantages and limitations. We offer a critical account of the variables which may contribute to inconsistent findings and the factors that should be considered when choosing a model and interpreting the results. We hope to present an insightful review of current AD mouse models and to provide a practical guide for selecting models best matched to the experimental question at hand.

## Background

**Fig. 1:**
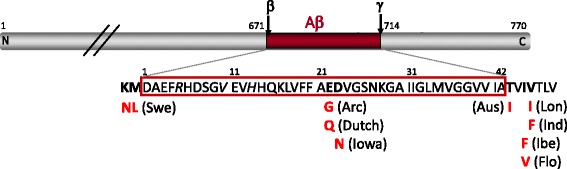
Diagram of APP illustrating nine FAD mutations that have been incorporated into mouse models. The Aβ domain is highlighted in red, with the β- and γ-cleavage sites identified at residues 671 and 714, respectively, using the numbering convention for full-length 770 amino acid protein. The amino acid sequence of Aβ is outlined in *red*, with the positions of several commonly used FAD mutations and their amino acid substitutions shown (in *bold*) alongside the geographic name identifying each variant. *Italic* residues indicate the three sites at which the Aβ sequence diverges between human and mouse (human is shown). Swe, Swedish; Arc, Arctic; Aus, Austrian; Lon, London; Ind, Indiana; Ibe, Iberian; Flo, Florida

Alzheimer’s disease (AD) is the most common form of age-associated neurodegenerative disorder clinically characterized by a decline in cognitive function and pathologically defined by the accumulation of extracellular β-amyloid (Aβ) plaques and intracellular neurofibrillary tangles (NFTs). Plaque Aβ peptide of 40 or 42 amino acids is produced by proteolytic cleavage of the amyloid precursor protein (APP) (Fig. 1), while NFTs are composed of hyperphosphorylated and misfolded tau protein. These neuropathological hallmarks are accompanied by profound neuroinflammation marked by astrocytic and microglial activation. A small percentage of AD cases are caused by genetic mutations in APP and presenilins identified in familial AD (FAD), where these mutations alter APP processing in favor of Aβ42 to drive the peptide aggregation thought to initiate disease. These genetic, biochemical, and neuropathological features form the basis for creating and validating animal models of AD. Beyond the autosomal dominant FAD mutations, the apolipoprotein E ε4 allele (APOEε4), along with rare point mutations in the triggering receptor expressed on myeloid cells 2 (TREM2), are strong risk factors for the more common sporadic, late-onset AD (LOAD).

Animal models are critical for understanding disease pathogenesis and also serve as valuable tools for preclinical testing. One of the most important considerations in working with rodent models is matching the mouse (or rat) to the experimental question under study. There are more than 100 different genetically engineered mouse lines reported to capture some aspect of AD - so many that it has become impossible to exhaustively track them all. We focus here on models that have been widely used and which remain available either privately or publically. We also highlight newly created lines that may be useful in modeling LOAD. The unavoidable first step in deciding among them is delineating the experimental question to be asked. Existing mouse models recapitulate amyloid plaques, neurofibrillary tangles, or neurodegeneration, but generally not all in the same mouse. Some models develop these hallmarks rapidly, others more authentically retain the aging facet of the disease. This review will describe key factors to consider when choosing between models, but you must know which features are essential for testing your hypothesis, as well as which are expendable. No mouse model is a faithful reproduction of human AD, but they can be useful tools when appropriately matched to the experimental question of interest.

### Matching your model to your experimental question - what is it that you want to study?

A myriad of models is at your fingertips. Most of them were designed to capture some aspect of disease pathology - and primarily plaques or tangles - with degeneration and cognitive decline emerging in some as serendipitous benefits. Choosing among them requires knowing what features of AD are required for your particular experiment, and so we describe below the main strategies that have been used to model these pathologies in genetically engineered mice.

#### Amyloid plaques and CAA

Amyloid plaques and cerebral amyloid angiopathy (CAA) both arise as insoluble deposits of the amyloid β peptide (Aβ). This peptide is derived by sequential cleavage of the amyloid precursor protein (APP) by the β-APP cleaving enzyme (BACE1) and γ-secretase at N- and C-termini respectively, to release three protein fragments: soluble APP (sAPP) and Aβ into the extracellular space and the amyloid-intracellular domain (AICD) into the cytoplasm. Mutations in APP were the first causes of early-onset FAD to be identified [1]; these autosomal dominant mutations tend to cluster around the β- and γ- processing sites to affect Aβ production. Transgenic expression of familial APP mutations provided the first successful means of reproducing amyloid pathology in mice [2] (Table 1); since then many dozen lines of APP-transgenic and knock-in have been created and characterized. Because amyloid deposition is time- and concentration-dependent, achieving pathology within the mouse lifespan requires that the production of Aβ be dramatically elevated relative to endogenous. This is most often accomplished by overexpressing human APP harboring one or more point mutations identified from FAD (Fig. 1). The Swedish mutation is most commonly used for this purpose (Swe), and is based on a two amino acid substitution adjacent to β-secretase cleavage at the N-terminus of Aβ [3]. The Swe mutation increases APP processing through the β-secretase pathway, thereby elevating production of Aβ relative to wild-type [4].

**Table 1:** Standard Transgenic Lines for APP, APP + PS1, and Tau

Model	Transgene	Transgenic promoter	Availability	Reference
*APP Transgenics*				
PDAPP	huAPP_770_ (Ind) minigene (cDNA + introns 6–8)	*PDGFB*	Novartis	[2]
Tg2576	huAPP_695_ (Swe)	hamster *Prnp*	Taconic, Charles River	[95]
C3–3	mo/huAPP_695_ (Swe)	mouse *Prnp*	MMRRC 34828-JAX	[96]
APP23	huAPP_751_ (Swe)	mouse *Thy1*	JAX 030504	[100]
J20	huAPP_770_ (Swe/Ind) minigene (cDNA + introns 6–8)	*PDGFB*	MMRRC 34836-JAX	[54]
TgCRND8	huAPP_695_ (Swe/Ind)	hamster *Prnp*	Peter St. George-Hyslop	[51]
Tg-SwDI	huAPP_770_ (Swe/Dutch/Iowa)	mouse *Thy1*	MMRRC 34843-JAX	[210]
*APP/PS1 Transgenics*				
APP/PS1 Line 85	mo/huAPP_695_ (Swe); Tg huPSEN1 (ΔE9)	mouse *Prnp*	MMRRC 34832-JAX, 34,829-JAX	[99]
APPPS1	huAPP_695_ (Swe); huPSEN1 (L166P)	mouse *Thy1*	Mathias Jucker	[211]
5XFAD Tg6799	huAPP_695_ (Swe/Flo/Lon); huPSEN1 (M146 L/L286 V)	mouse *Thy1.2*	MMRRC 34840-JAX, 34,848-JAX	[47]
*Tau Transgenics*				
Tau Tg Line 43	huMAPT_3R0N_ (wt)	mouse *Prnp*	JAX 003741	[212]
JNPL3	huMAPT_4R0N_ (P301L)	mouse *Prnp*	Taconic	[34]
hTau.P301S	huMAPT_4R0N_ (P301S)	mouse *Thy1.2*	Michel Goedert	[213]
PS19	huMAPT_4R1N_ (P301S)	mouse *Prnp*	JAX 008169	[33]
*APP/PS1/Tau Transgenic*				
3xTg-AD	huAPP_695_ (Swe); MAPT_4R0N_ (P301L); *Psen1* ^M146V^ knock-in	mouse *Thy1.2*	MMRRC 34830-JAX	[101]

Tables 1-4: Refer to Fig. 1 for illustration of APP mutations.

Additional mutations may also be incorporated at the γ-secretase C-terminus of Aβ to elevate the ratio of Aβ42 relative to the predominant but less aggregation-prone Aβ40. Several familial C-terminal mutations have been used in mouse models including those identified from Indiana (Ind, V717F [5]), London (Lon, V717I [1], Florida (Flo, I716V [6]) and the Iberian Peninsula (Ibe, I716F [7]). By elevating the ratio of Aβ42:40, these C-terminal mutations enhance Aβ aggregation and accelerate the formation of amyloid plaques to produce early onset pathology. Plaque onset can also be accelerated through familial mutations in presenilin 1 (*PSEN1)*, such as M146V [8], M146L [9], L166P [10], L286V [8], or exon 9 deletion (dE9 [11]) (see http://www.alzforum.org/mutations for details). These PSEN1 mutations shift APP processing by γ-secretase to produce longer, more pathogenic Aβ peptides which are useful for accelerating disease in short-lived animal models.

A handful of models also include APP mutations within the central Aβ domain that foster vascular amyloid accumulation. Unlike the β- and γ- cleavage site mutations used to boost Aβ production or shift the 42:40 ratio without affecting the Aβ sequence itself, these internal mutations change the amino acid sequence of Aβ. The mutations most often used in this manner were identified from kindreds in the Netherlands (Dutch, Aβ E22Q [12, 13]), Arctic (Arc, Aβ E22G [14, 15]), and Iowa (Aβ D23N [16]).

In all cases, whether APP modifications are introduced through an over-expressed transgene or via targeted modification of the endogenous allele (knock-in, Table 2), the amino acid sequence of Aβ itself is almost always converted from mouse to human by 3 amino acid substitutions at Aβ residues G5R, F10Y and R13H. BACE1 processes murine APP at the +11 rather than +1 site of Aβ [17]. As a result of this shift, mouse Aβ11–40/42 does not aggregate in vivo [18]. Simply humanizing the Aβ sequence yields full-length 1–40/42 peptide which readily aggregates in a time- and concentration-dependent manner to produce amyloid deposits.

**Table 2:** Lines with physiological expression of APP, PS1, and Tau

Model	Allele	Targeting approach	Availability	Reference
APP^NLh^	endogenous mouse *App* with Swe mutation and humanized Aβ domain	Knock-in	NA	[89]
APP R1.40	complete human genomic *APP* (Swe)	YAC transgenic	MMRRC 34831-JAX	[86]
APP^SL^	mouse/human *App* hybrid with humanized Aβ domain (Swe/Lon)	Knock-in	NA	[92]
APP^DSL^	endogenous mouse *App* with humanized Aβ domain (Swe/Dutch/Lon)	Knock-in	Hui Zheng	[57]
APP^NL^, APP^NLF^, and APP^NLGF^	endogenous mouse *App* with humanized Aβ domain and (Swe (NL), Swe/Ibe (NLF), or Swe/Arc/Ibe (NLGF))	Knock-in	Takaomi Saido	[58]
PS1^M146V^	endogenous mouse *Psen1* (M146 V)	Knock-in	JAX 004193	[214]
PS1 KI	endogenous mouse *Psen1* (P264L)	Knock-in	NA	[215]
htau	complete human genomic *MAPT* (wt) with targeted disruption of murine *Mapt*	PAC transgenic x *Mapt* KO	JAX 005491	[37]

Tables 1-4: Refer to Fig. 1 for illustration of APP mutations

The amyloid plaques and CAA produced by genetic modification of APP in mice share many characteristics of the human neuropathology, but important distinctions exist. These distinctions have been recently reviewed and are therefore not covered here in any detail [19]. Generally speaking, most APP modified mice develop both diffuse and fibrillar Aβ deposits that can be distinguished by comparing silver staining or Aβ immunohistochemistry against Congo red or thioflavin-S histology. Fibrillar plaques are often surrounded by reactive astrocytes and microglia and by dystrophic neurites (axonal swellings). Synapse loss can be observed in the zone immediately adjacent to fibrillar deposits and several models display mild neuron loss with age, but none develop the severe atrophy observed in human AD. Some APP models develop modest levels of hyper-phosphorylated tau but none develop true neurofibrillary tangles seen in human AD.

#### Neurofibrillary tangles

Neurofibrillary tangles (NFTs) composed of aggregated tau protein are a pathological hallmark of AD and more than 20 other neurodegenerative conditions collectively known as tauopathies. The severity of NFTs correlates better than amyloid plaques with cognitive decline and neurodegeneration in AD [20]. Under normal physiological conditions, tau is an unstructured axonal protein that binds and stabilizes microtubules [21]. Hyper-phosphorylation and other aberrant post-translational modifications lead to misfolding and dissociation from microtubules. Redistribution of misfolded tau into the soma and apical dendrites generates NFTs; redistribution to distal dendrites yields neuropil threads [22]. NFT pathology progresses in a hierarchical, stereotyped pattern beginning in the transentorhinal cortex, and gradually extending to hippocampus before ultimately reaching other cortical areas. This pathological spreading through synaptically connected regions is the basis of Braak staging in AD [23]. Mounting evidence from cell culture and mouse models supports the cell-to-cell transfer of tau pathology [24].

In humans, tau is expressed as both three-repeat (3R) or four-repeat (4R) isoforms due to alternative splicing of exon 10 in the microtubule-associated protein tau gene (*MAPT*), however, only 4R tau is expressed in adult mice. Mutations in *MAPT* are not found in AD, but instead cause a subtype of frontotemporal lobar degeneration (FTLD), demonstrating that this pathology is sufficient for neurotoxicity and dementia [25]. Both coding and non-coding mutations have been identified; many of these affect MAPT splicing to favor the 4R isoform arguing that the balance of 3R/4R is important for neuronal health [26, 27]. The genetic imbalance associated with these *MAPT* mutations in FTLD raises the question of whether isoform dysregulation may also underlie sporadic tauopathies such as AD. Based on these genetic findings, multiple transgenic mouse lines have been created to overexpress human 4R tau containing FTLD mutations. Of these, the two models, rTg4510 and PS19, are most commonly used.

rTg4510 is a bigenic line in which the human 4R tau with the P301L mutation is expressed in forebrain neurons under indirect control of the *Camk2a* promoter (described in more detail below under “*Controllable transgenics*”). The mutant tau transgene is expressed at levels ~13-fold higher than endogenous mouse tau and the mice develop aggressive NFT pathology and neurodegeneration [28, 29]. Pathological forms of phospho-tau can be observed at 2–3 months of age followed by mature tangles in the cortex at 4 months and hippocampus at 5.5 months. The model is based on the tetracycline-transactivator expression system and therefore affords the flexibility of turning the transgene on and off (again, see “*Controllable transgenics”* section for details); the tau P301L responder line can also be crossed with other transactivator lines, e.g. EC-tTA [30], to drive expression of mutant tau in other parts of the brain [31, 32]. This flexibility comes with a cost: the system requires two transgenes for expression which complicates further genetic crosses.

The PS19 model is a traditional (non-controllable) transgenic line in which the human 4R tau with the P301S mutation is controlled by the mouse prion promoter, resulting in ~5-fold overexpression compared to the endogenous mouse tau [33]. The tau phenotype is considerably milder than that of rTg4510, with the onset of phospho-tau pathology at ~6 months. Of note, these mice develop hindlimb paralysis due to transgene expression in spinal cord and die between 10 and 12 months of age depending on the genetic background. As a result, mature, thioflavin-S positive NFTs are rare. These same limitations in neuropathology and early lethality are also seen in the JNPL3 line expressing 4R P301L tau under the mouse prion promoter [34].

Generally speaking, overexpression of mutant tau is required for phospho-tau and NFT pathology to manifest. Contrary to most APP/Aβ models and consistent with human clinicopathology, the tau mice exhibit age-dependent neurodegeneration in addition to synaptic and cognitive deficits, suggesting that misfolded and/or aggregated tau is directly neurotoxic. The onset and severity of pathology largely correlate with the level of transgene overexpression and the mutation used. Both the rTg4510 and PS19 mice are suitable models for testing the pathological consequences of tau accumulation. Due to its early onset, rTg4510 serves as a good model to test manipulations expected to ameliorate pathology but not those expected to accelerate; the PS19 model can be used for both purposes. The PS19 mice have also been widely used to model “tau transmission” by inoculating either purified tau fibrils or brain lysates from tauopathy patients or tau transgenic mice, and tracking the appearance of phospho-tau in synaptically connected regions over time [35, 36].

While the tau transgenic models have been successful in modeling NFT pathology and functional impairment, it is important to recognize their shortcomings. First, since no tau mutations have been linked to AD, these mice should be considered models of FTLD, not AD. Second, the transgenic mice express tau cDNA and do not afford modulation of alternative splicing and 3R/4R ratio. Finally, ectopic expression of transgenic tau may not recapitulate the natural evolution of NFT spreading through synaptically connected regions. The htau mice described below overcome these limitations.

Duff, Davies and colleagues created a humanized tau (htau) model by expressing wild-type human *MAPT* genomic DNA on a mouse *Mapt* knockout background [37]. These mice preserve the native human 3R/4R ratio and were initially described to show insoluble, hyper-phosphorylated tau by ~9 months of age with spatiotemporal progression resembling early stages of human AD. The htau mice also developed age-dependent synaptic and behavioral deficits as well as neuronal loss [38]. These features make htau an attractive model that is pathophysiology relevant to AD. Nevertheless, some important caveats should be noted. The behavioral and neurodegenerative phenotypes appear to be very mild and may not be readily reproducible [39, 40]. Although not verified, it is suspected that the increasingly delayed and mild phenotypes observed since the model was first introduced may result from shortening of the transgene array over generations of breeding, informally known as ‘copy dropping’. The mild phenotype necessitates testing at advanced ages, with the attendant complications in variability, lethality, and cost. Finally, the presence of two alleles (human *MAPT* transgene and mouse *Mapt* deletion) complicates additional genetic manipulation.

#### Neurodegeneration

The strongest models of AD-related neurodegeneration come from transgenic overexpression of *MAPT* mutations associated with FTLD. In particular, the rTg4510 model expressing P301L tau develops severe forebrain atrophy, losing up to 40% of gross brain weight and >65% of CA1 pyramidal neurons by 5.5 months of age [28, 29, 41]. Significant forebrain atrophy with loss of cortical and hippocampal volumes is also observed in the P301S tau model PS19 at advanced ages [33]. In both of these tau models, neuronal loss outside of the hippocampus also observed. Where quantified for the rTg4510 model, neuron loss in cortex begins later than in hippocampus but still reached >50% by 8.5 months [41]. Even the htau model overexpressing wild-type human *MAPT* was initially described with notable neuronal loss, albeit at much later ages (~17 months) [42], and may have since waned with successive generations of breeding (see “*Neurofibrillary tangles*” section for more detail).

Only four models of amyloid pathology have shown any substantial degree of neuronal loss, and none of the APP mutations produce the same degree of neurodegeneration in mice that is observed in humans. The first APP mutation model described with neuronal loss was APP23, in which the number of hippocampal CA1 pyramidal cells decreased 14–25% by 14–18 months of age, commensurate with plaque load [43]. Neuronal loss was specific for the hippocampus, however, and neuron counts were unchanged in the neocortex of this model [44]. The second model found to develop significant neuronal loss was an intercross between transgenic APP^Swe/Lon^ and PS1^M146L^ lines, which displayed ~25% loss of CA1 pyramidal neurons by 17 months of age [45]. The same group described even greater loss of CA1 neurons in a second APP x PS1 model later that year [46]. This latter model combined 4 FAD mutations (APP Swe + Lon and PS1 M233 T + L235P) to evoke ~ 50% loss of CA1 neurons by 10 months of age. Intriguingly, cell death was again specific to CA1 hippocampal neurons with no net decline in either CA3 or dentate gyrus. The most recent and perhaps most widely used amyloid-based model in which neuronal loss has been described is the 5XFAD line. This mouse is named for its incorporation of 5 distinct AD mutations into a single transgenic line (APP Swe + Flo + Lon and PS1 M146 L + L286 V), which collectively produce rapid amyloid pathology as early as 2 months of age [47]. Neuron loss has been qualitatively described in the subiculum by 9 months of age; in the neocortex, non-biased stereological estimates suggest that 5XFAD mice lose 25–40% of layer 5 pyramidal neurons between 9 and 12 months of age [48, 49]. Neuron loss has not been described in other cortical layers, and despite the marked decrease in layer 5 neurons, the mice show no change in the total number of cortical neurons [48]. This outcome is consistent with the absence of overt brain atrophy in this and other models of Aβ amyloidosis.

#### Cognitive impairment

A surprising number and variety of mouse AD models develop some form of cognitive impairment. Where they have been tested, nearly all models of Aβ overproduction based on transgenic APP overexpression show deficits in spatial learning and memory (reviewed in [50]). In many but not all APP models, the onset of cognitive decline occurs in close proximity to that of amyloid deposition, such as in the CRND8 model [51], but has been identified both months prior to pathology in the Tg2576 model [52, 53] and months afterwards in the 5XFAD [47] and tet-off APP models (Chiang et al., in press). Of note, the age and disease stage at which cognitive deficits are observed depends in part on the task being used. This is well illustrated by behavioral characterization of the J20 model: this line shows initial plaque onset by 6 months of age [54], but when tested at 12–16 months performed as well as age-matched controls in the Y-maze and contextual fear conditioning despite showing severe impairment in a cheeseboard test of spatial memory [55].

Even knock-in models based on modification of the endogenous *App* sequence alone or in addition to mutation of *Psen1* can develop learning and memory impairment with age (i.e., homozygote APP^NLh^/PS1^P264L^ [56] or APP^NLh^/PS1^M146V^ [39, 57]. Perhaps to an even greater extent for knock-in models than for transgenics, both age and task can influence the reproducibility and robustness of cognitive phenotypes. This caveat is especially poignant when considering the newest set of APP knock-in models (i.e., APP^NL^, APP^NLF^, and APP^NLGF^) where behavioral deficits in the same mouse line have varied between laboratories [58]. Using automated IntelliCage testing equipment, Masuda et al. detected only mild deficits in learning, recall, attention, and cognitive flexibility for all but the most aggressive homozygote APP^NLGF^ line [59]. However, a follow up study of the APP^NLGF^ model detected changes in locomotor/exploratory activity, but not learning and memory, despite widespread amyloid deposition [60]. This example serves as a good reminder to check that the model you intend to study has a phenotype in the outcome you intend to measure.

Models of neurofibrillary pathology also show varying degrees of cognitive impairment depending on age, task, and transgene. Like the amyloid models, the tau transgenic lines with more aggressive pathological phenotypes also tend to show more pronounced cognitive changes. The aggressive rTg4510 model develops progressive worsening in spatial memory that parallels the onset of tau hyper-phosphorylation and neuron loss [28, 29]. Similar deficits, although not as clearly progressive in nature, have been reported for the PS19 tau model [61–63]. Learning and memory impairments are less consistent for tau models with milder pathological phenotypes. For example, the htau model expressing human wild-type tau on a mouse tau null background was initially reported to show age-related decline in spatial learning and object recognition memory [38], however, more recent studies have not reproduced these deficits [39, 40, 64]. Variable cognitive impairment has also been reported for the JNPL3 mouse which expresses near-endogenous levels of P301L tau [65, 66]. Our ability to detect progressive learning and memory impairment in mice is limited by both the sensitivity and the range of the behavioral tasks we have available. The take home message may be that the chances of observing consistent and reproducible changes in cognitive function against which to gage effects of therapeutic intervention or genetic manipulation are increased by choosing a model that develops substantial AD pathology, regardless of whether this comes in the form of plaques or tangles.

### Yin and yang of mouse models: Deciding between transgenics and knock-ins

After deciding what experimental question you want to address and which phenotype your model must recapitulate to answer that question, you must next decide whether a transgenic mouse line will suffice or if a knock-in model is needed. The central difference between these two approaches is the pattern of protein expression used to induce disease phenotype. In standard transgenic lines, a synthetic cDNA often encoding a disease-associated mutation is controlled by a heterologous promoter that results in an artificial expression pattern of limited spatial and temporal fidelity to the endogenous gene. In knock-in models, the native expression pattern is fully preserved but the protein now contains a disease-associated mutation (i.e., APP^Swe^) or human-specific variant (i.e., APOEε4). Standard transgenics typically involve biased overexpression of a single splice variant, while knock-ins preserve native splicing at physiological levels. While it is intuitively preferable to use a model that most closely approaches endogenous expression patterns, there are situations where this is not possible or appropriate and where a transgenic model would do better, particularly in driving robust disease pathology. Knowing which compromises can be tolerated is just as important as knowing which may confound your experimental outcome.

There are many cases where the artificial expression of a single disease-associated protein variant may be the most efficient or only way to produce a phenotype in mice. Currently, overexpression of mutant human 4R tau is the only approach that has succeeded in generating thioflavin- and silver-positive neurofibrillary tangles in mice [29, 33]. Interventions to slow tangle formation therefore often accept the compromise between the presence of pathology and the artificial means by which it was produced (for example, [63, 67]). The use of transgenic models is also a reasonable compromise in studies testing cell non-autonomous effects of protein aggregation, where the cells under study are essentially wild-type (for example, [68]).

In contrast, transgenic overexpression can be problematic for studies where the effects of artificial temporal or spatial expression confound the outcome. For example, we found that transgenic APP expression under control of the *Camk2a* promoter caused persistent locomotor hyperactivity that prevented accurate cognitive testing [69]. The effect was traced to a consequence of transgenic APP on circuit development when we found that delaying transgene expression until adulthood was sufficient to abate the locomotor phenotype [70]. The same neurodevelopmental interaction also produced cortical hyper-synchrony in young adult APP mice that could again be abated by delaying transgene onset [71]. Although we found a workaround that met our needs, the emergence of knock-in models preserving the endogenous pattern of APP expression would avoid this confound entirely.

Another factor to bear in mind is that the endogenous proteins overexpressed in transgenic models have physiological functions independent of their role in Alzheimer’s pathology. For example, overexpression of APP increases the production of soluble APP (N-terminal) fragments, membrane-associated C-terminal fragments, and the cytoplasmic APP intracellular domain (AICD) in addition to Aβ. Endogenous APP and its fragments contribute to cellular adhesion, neurite outgrowth, synapse formation, neuronal survival, and intracellular signaling [72–75]. Thus it is conceivable and perhaps likely that some of the phenotypes observed in APP transgenic mice arise from over-activation of these innate functions [76]. Along with the differences in spatiotemporal expression, elevated levels of these APP fragments may account for some of the reported distinctions between transgenic and knock-in models. The same concerns will also apply to tau transgenic models, where overexpression of a single isoform also creates an imbalance of the endogenous splice variants.

Reassuringly, several disease-relevant phenotypes identified in APP transgenic animals have been reproduced in knock-in models, including Aβ plaque formation, microglial and astrocytic activation, loss of synaptic markers, impaired hippocampal neurogenesis and diminished gamma frequency oscillations [58, 76–79]. Other features, such as calpain activation, show less agreement between transgenic and knock-in mice [80]. Of note, cognitive impairment that has been described in numerous APP transgenic models [50] has been more variable in knock-in models, albeit far less well studied [56, 58–60]. Phenotypic agreement and discrepancies are also found in the comparison of tau transgenic mice with the htau model which is currently the nearest available to a true knock-in. Admittedly, the comparison between transgenic tau mice expressing mutant variants and htau mice expressing wild-type protein is less apt than for APP models where both transgenics and knock-ins carry FAD mutations. Nevertheless, multiple phenotypes identified in tau transgenic models have also been reported in the htau mouse, including tau hyper-phosphorylation [37, 39, 42, 81–83], diminished neuronal integrity and plasticity [38, 84, 85] and elevated tau kinase activity [42]. Disparities between tau transgenics and the htau model are also observed, most notably in the extent of neurodegeneration, gliosis, and cognitive decline which are quite limited and/or absent even in aged htau animals [39, 40, 81], but see [38, 42].

Taken together, the best approach to mitigate against outcomes arising from the compromises inherent in transgenic models is to test the same outcome in multiple complementary lines. Because the cost of animal studies often renders this impractical, a reasonable alternative might be to choose the model with closest construct validity to the human disease that recapitulates the phenotype under investigation. Perhaps it is easier said than done, but where possible, use models that maintain endogenous protein expression patterns, where not, recognize the caveats that necessarily attend any interpretation of the resulting data.

### Factors to bear in mind with knock-in models

While the bulk of early work to model AD in mice was done by standard transgenesis, a parallel thrust in the field sought to build models which avoided ectopic overexpression and maintained native transcript variation. The earliest of these models introduced the entire human *APP* or *MAPT* genes and their regulatory elements into mice via yeast or P1-derived artificial chromosomes (YAC or PAC vectors, respectively) [86–88]. While these models achieved native expression patterns and isoform usage, they shared with traditional transgenic mice the potential for endogenous gene disruption by random chromosomal integration. In addition, the murine *App or Mapt* gene retains expression and may interfere with the human transgene. Evidence for such a cross-species interaction emerged during construction of the htau model where hyper-phosphorylated tau was only observed once the human MAPT PAC mice were placed on a *Mapt* null background [37]. Due to the near-endogenous expression level, the APP YAC mice only develop measurable Aβ pathology when a FAD mutation is included (i.e., Swe) and the transgene is bred to homozygosity. Despite their benefit in construct validity, the mild phenotypes and complicated genetic crosses have limited use of the APP YAC and htau models.

An alternative strategy to achieve physiological expression is the knock-in (KI) approach, which is used to modify selected genes without perturbing off-target genomic perturbations. Multiple *App* and *Psen1* KI lines have been created, but no *Mapt* lines have been reported to date (Table 2). The first APP KI line, APP^NLh^, reported in 1996 humanized the Aβ sequence and introduced the Swedish mutation into the mouse *App* gene [89]. Despite these modifications, the APP^NLh^ model did not develop Aβ pathology or other overt anomalies until interbred with the PS1^P264L^ knock-in line to produce amyloid deposits, synaptic impairments, and altered hippocampal neurogenesis [79, 90, 91]. Additional *App* knock-in lines have since been created testing various FAD mutations in an effort to promote Aβ pathology. These include APP^SL^ expressing the Swedish and London mutations [92], APP^DSL^ expressing the Swe, Lon, and Dutch mutations [57], and the recent allelic series from Saido and colleagues expressing the Swe (APP^NL^), Swe and Beyreuther/Ibe (APP^NLF^), or Swe, Arc, and Ibe mutations (APP^NLGF^) [58], all with a humanized Aβ domain. Like the original APP^NLh^ model, homozygous APP^SL^ and APP^DSL^ mice failed to develop Aβ pathology unless interbred with *Psen1* mutant mice. However, unlike the APP^NLh^ and APP^SL^ models, addition of the Dutch mutation in the APP^DSL^ mice promoted formation of vascular amyloid characteristic of cerebral amyloid angiopathy (CAA), with cerebral blood flow reduction, late-onset microhemorrhage, and cognitive impairment - but only when intercrossed with a *Psen1* mutant line [57].

Unlike the Lon mutation, the Ibe mutation is sufficient to produce Aβ deposition when combined with the Swe mutation in homozygous APP^NLF^ knock-in mice; addition of the Arctic mutation in APP^NLGF^ mice accelerates this phenotype [58]. These models take advantage of the exceptionally high Aβ42 production afforded by the Beyreuther/Ibe mutation to generate pathology in the absence of *Psen1* mutation. Indeed, the Aβ42/40 ratio was 30-fold higher than under physiological conditions. Similar to other amyloid models, homozygous APP^NLF^ and APP^NLGF^ mice develop age-dependent loss of synaptic markers and reactive gliosis, but show more limited behavioral phenotypes (see *“Cognitive impairment”* section for details) [58, 59].

Overall, the *App* knock-in models provide precise genetic manipulation and faithful physiological expression of target genes. Achieving this physiological fidelity necessitates several genetic compromises for amyloid pathology to manifest. For example, all amyloid-forming knock-in mice carry multiple FAD mutations and in most cases must be bred to homozygosity to elicit Aβ pathology. Additionally, several earlier *App* knock-in lines required homozygous expression of mutant *Psen1* alleles to obtain amyloid deposition (APP^NLh^, APP^SL^ and APP^DSL^), while others do so through supra-physiological production of Aβ42 (APP^NLF^ and APP^NLGF^). It is also important to note that the Dutch and Arctic mutations used in the APP^DSL^ and APP^NLGF^ mice change the primary sequence of Aβ to influence its biophysical properties [15, 93]. These technical and biological issues should be taken into consideration when deciding whether a knock-in model is best for your experimental needs, and if so, which line to use.

### Technical considerations for transgenic models

To an even greater degree than for knock-in models, multiple design factors go into creating each transgenic line and should be considered when choosing a model for study. The central disease feature to be examined, be it plaques, tangles, degeneration, or cognitive decline, will determine which class of models to consider, but within that class, the best model may depend on a variety of other factors such as where and when critical features of disease will appear. Simply put, the best model isn’t always the one you can get from the lab down the hall.

#### The transcript itself

Most transgenic constructs are based on artificial cDNAs designed to express a single transcript out of many that are produced by alternative splicing of the endogenous gene. One example of this is the frequent use of the 695 amino acid isoform of APP, the shortest of three alternatively spliced variants (along with 751 and 770 [73]). Because the 695 isoform is predominantly or perhaps exclusively expressed in neurons and accounts for most APP in the brain [94], its use in transgenic constructs intended to increase Aβ production in the brain has been a logical strategy in many cases (i.e., Tg2576 [95], C3–3 [96], and TgCRND8 [51]). Another approach taken in some early transgenic models was to create an APP minigene containing all 18 exons and capable of producing the three main isoforms of 695, 751, and 770 amino acids (i.e., PDAPP [2, 97], and J20 [54]). Both approaches have yielded amyloid pathology in the mouse brain.

Most tau transgenic models have also been constructed using cDNA derived from just one of the six transcripts for human *MAPT*. Several tau models incorporate a familial mutation from FTLD located in the alternatively spliced exon 10 and therefore produce only 4R tau protein rather than a mixture of 3R and 4R tau (i.e., rTg4510 [29] and PS19 [33]). In an effort to overcome the artificial bias introduced by transgenic production of a single tau isoform, Duff and colleagues used a plasmid artificial chromosome (PAC) to introduce the entire human wild-type *MAPT* gene and upstream regulatory sequence into the mouse (i.e., Line 8c [87]). These mice produce all 6 isoforms of human tau at levels several-fold higher than endogenous, but do not develop pathological hyper-phosphorylation unless murine tau is eliminated [37]. In the case of AD, it is known that both 3R and 4R tau protein contribute to pathologic aggregates [98], but the success of 4R transgenic models suggests that this disease phenotype can be reasonably modeled using just one transcript. In other experimental settings, native transcript variation is required. The nature of the experimental question will dictate whether models based on a single transcript are an acceptable substitute in each case.

#### The transgenic promoter

Historically, the promoter elements used in transgenic constructs were chosen to ensure robust, widespread, life-long expression of ectopic protein. Several common promoter constructs have been used over the years, including prion protein (*Prnp*, i.e., Tg2576 [95], TgCRND8 [51], APP/PS1 Line 85 [99], JNPL3 [34], and PS19 [33]), platelet-derived growth factor B chain (*PDGFB*, i.e., PDAPP [2] and J20 [54]), and thymus cell surface antigen 1 (*Thy1*, i.e., APP23 [100], 5XFAD [47], and 3xTg-AD [101]). While these three promoters induce strong and persistent transgene expression in neurons, *PDGFB* and *Prnp* are also active to a lesser extent in non-neuronal cells of the CNS [102], and all three promoters elicit expression in multiple organs outside of the nervous system including heart and liver [103–105]. All three promoters are active in the embryonic brain [103, 105, 106], and in the adult may be expressed in multiple neuronal subtypes [107–112]. Although these promoters were chosen for strong persistent expression in adult neurons, none of the three is restricted to the CNS and all are active embryonically. If the experimental question at hand requires a more selective temporal or spatial expression pattern, alternative genetic strategies such as the controllable systems described below will be needed to achieve this specificity.

#### Strain background

In the past, most transgenes were made using hybrid mouse strains such as C3B6 (APP/PS1 Line 85 [99, 113] and PS19 [33]), B6D2 (J20 [54]) or B6SJL (Tg2576 [95]). The push towards congenic strain backgrounds lead to many lines being backcrossed onto a single parental strain, revealing the impact that genetic context could have on transgene-associated phenotypes. Early work on the Tg2576 model revealed that the transgene was well tolerated on the original hybrid background but caused early lethality when backcrossed to C57BL/6 [114]. By the fourth generation, none of transgenic offspring survived past 2.5 months of age. Other APP^Swe^ transgenic lines have since been backcrossed to C57BL/6 for >10 generations without loss (i.e., APP/PS1 Line 85, C3–3, and J20), suggesting that this strain is not inherently problematic for AD models and that the premature lethality of Tg2576 likely arose from a specific interaction between the genomic integration site and genetic modifiers of the background strain. Later work using the YAC transgenic model R1.40 demonstrated that genetic background could influence both APP processing and the age at which amyloid deposits appeared, dramatically delaying onset from 13.5 months on C57BL/6 to >20 months on DBA/2 [115]. These two background strains also influenced phenotype in the APP/PS1 line 85 mice, where the DBA/2 background substantially increased susceptibility to lethal seizures compared to C57BL/6 [116]. Multiple genetic loci may contribute to these strain differences [117], including the kinesin light chain-1 gene identified as a modifier of amyloid onset in the DBA/2 background [118]. As these studies demonstrate, the genetic context in which any transgene acts can significantly influence the resulting phenotype. Several transgenic models are available on multiple inbred and hybrid backgrounds (5XFAD, APP/PS1 Line 85, Tg2576), and it is worth investing some time into weighing the options between experimental needs and known characteristics of each strain (i.e., FVB/N breed well but are blind as adults; C57BL/6 mice breed less well but are a reasonable option for cognitive testing, etc.).

#### Age of onset

The age at which disease-associated phenotypes first appear in each model results from a complex mixture of the strength of the transgene promoter, the aggressiveness of familial mutations included in the transgene, the number of transgene copies incorporated into the insertion site, the chromosomal location of transgene integration, and the genetic background on which the transgene is expressed. The design of the transgene construct likely has the greatest impact on the level at which the transgene is expressed and thus on the age at which phenotypes appear, however, chromosomal integration site and copy number also have substantial influence and cannot (usually) be controlled. As an example of how these latter factors can affect onset, TgCRND8 and C3–3 models both express APP^Swe^ under control of a prion protein promoter, yet amyloid pathology appears by 2 months of age in TgCRND8 but not until 18 months in C3–3 [51, 119]. If the experimental manipulation is predicted to delay pathogenesis, then an early onset model may be most appropriate. Conversely, if acceleration is anticipated, a late onset model would be better. Bear in mind, however, that while the early onset models may be faster to study, they also create a disconnect between chronological age and pathological condition that does not accurately reflect the aging physiology under which most AD will occur.

#### Animal source

Although it is easy and inexpensive to obtain transgenic models from a lab down the hall, publically-supported repositories like the Jackson Laboratory (Jax) or the Mutant Mouse Research and Resource Centers (MMRRC) in the US, the RIKEN BioResource Center (BRC) Experimental Animal Division in Japan, and the European Mouse Mutant Archive (EMMA) provide genetic validation of their models that is well worth the added cost. As an example of this, Jax screens all incoming models for the presence of common extraneous alleles (Cre, Flp, neo, tTA, GFP and RFP), often to discover that donating labs have intercrossed the line with another transgenic resulting in mistyped offspring that continue to carry the unrecognized modification. Once identified, these extraneous alleles can be removed before the strain is cryopreserved or expanded for distribution. Moreover, Jax routinely genotypes new mice using a single nucleotide polymorphism (SNP) panel to characterize the strain background and elucidate any uncertainties in the parentage of the donated animals. The stock or strain number of the transgenic line also provides a clear means of identifying which model was tested. Common nomenclature can be muddied by varying abbreviations adopted by different laboratories, therefore the stock number can be a universal identifier easily referenced by other researchers. Finally, by supporting communication with receiving investigators, issues that arise in the field can be verified and shared, as done recently for the 3xTg-AD mice upon learning from the donating investigator that male mice may no longer display the phenotypes initially described for this line, while female animals appear unaffected (https://www.jax.org/strain/004807). Similar reporting from users in the field lead to the identification of transgene copy loss in the J20 strain that was remedied by re-importing the line from the donating laboratory; qPCR is for copy number is now a routine part of colony maintenance for this strain (https://www.jax.org/strain/006293). This type of unpublished information and quality assurance are invaluable in helping to ensure that research effort and resources yield informative and reproducible outcomes. Don’t waste your time or funding on mice that aren’t what they’re meant to be.

#### Sex as biological variable

In general, female mice are more susceptible to plaques and tangles than their male counterparts. Earlier-onset pathology has been consistently noted in females across multiple APP transgenic models, including Tg2576 [120], APP/PS1 [121, 122], an intercross of APP^Swe^ x PS1^A246E^ [123], and 3xTg-AD [124]. Where tested, transgene expression appears similar in male and female APP mice [125]; instead the accelerated pathology may be attributable to increased β-secretase processing in females [124, 126]. Tau pathology also tends to be more pronounced in female transgenic mice, as noted in the rTg4510 [127] and JNPL3 models [128, 129], albeit not in 3xTg-AD [124, 130]. Perhaps as a result of elevated neuropathology, cognitive impairments are also generally greater in female mice, including Tg2576 [131], APP/PS1 [121], CRND8 [132], APP/TTA [125], 3xTg-AD [130], and rTg4510 models [127]. Females also respond more drastically to environmental stress, developing higher amyloid levels in 5XFAD [133], insoluble and caspase-cleaved tau in a tau P301L model [134]. Not all phenotypes are female-biased, however, as markers of neuroendocrine aging appeared earlier in male than female 3xTg-AD mice [135]. While sex-based differences in pathological phenotypes are not found in every model and are not consistent even for a single feature across all lines, there is accumulating evidence that it can make a difference and should be considered when designing experiments and measuring outcomes. The NIH has made a strong case for greater attention to sex as a biological variable in all basic and translational research, and it is clearly an important factor to consider for AD where gender clearly contributes to risk [136].

### Alternative approaches for transgene expression - viral gene delivery

While genetically engineered mice are the most common animal models for AD, viral-mediated gene expression systems have been increasingly used to elicit neuropathology either alone or in conjunction with existing genetic models. Two viral systems have been employed for gene expression in rodent brains: lentivirus and adeno-associated virus (AAV). While both systems deliver localized gene expression when stereotaxically injected to the adult brain, AAV can also be used to achieve widespread gene expression when injected into the neonatal brain. There are at least 13 different AAV serotypes with varying tropism. Of these AAV serotypes 1, 2, 6, 8, 9, and 10 have been demonstrated to drive neuronal expression of target genes. Table 3 summarizes the viral models of Aβ and tau pathology created using wild-type rat and mouse.

**Table 3:** Viral transgenic lines for APP and Tau

Transgene	Promoter	Viral packaging	Host	Reference
*Stereotaxically Targeted APP/Aβ Viral Transgenics*				
BRI-Aβ40 BRI-Aβ42	CBA	AAV1	adult rat	[137]
huAPP_695_ (Swe/Lon/Aus)	*(human?) EF1A*	AAV (serotype not stated)	adult mouse	[138]
*Stereotaxically Targeted Tau Viral Transgenics*				
hu*MAPT*_4R2N_ (P301L)	CAG	AAV2	adult rat	[142]
hu*MAPT*_4R0N_ (P301S)	mouse *Pgk*	lentivirus	adult mouse	[141]
hu*MAPT*_4R1N_ (P301L)	CMV	AAV2	adult mouse	[216]
hu*MAPT*_4R2N_ (P301L) hu*MAPT*_4R2N_ (wt)	human *SYN1*	AAV1	adult mouse	[217]
hu*MAPT*_4R1N_ (P301L) hu*MAPT*_4R1N_ (wt)	CMV	lentivirus	adult rat	[140]
hu*MAPT*_4R2N_ (P301L)	human *SYN1*	AAV9	adult mouse	[218]
hu*MAPT*_4R0N_ (P301S) hu*MAPT*_4R0N_ (wt)	mouse *Pgk1*	AAV6	adult mouse	[219]
*Whole-Brain Tau Viral Transgenics*				
hu*MAPT* (P301L) (isoform not stated)	CAG	AAV1	neonatal mouse	[143]
hu*MAPT*_4R2N_ (P301L)	CAG	AAV1	neonatal mouse	[220]

*CAG* cytomegalovirus immediate early enhancer combined with the chicken β actin promoter [209]

*CBA* chicken β-actin

*CMV* cytomegalovirus

Tables 1-4: Refer to Fig. 1 for illustration of APP mutations.

Viral expression of APP has not yielded much success in producing robust amyloid pathology. Out of a series of amyloid-based expression vectors injected into the hippocampus of adult rats (Aβ40, Aβ42, and APP^Swe^), amyloid deposits were only detected in animals receiving Aβ42 virus (i.e., BRI-Aβ42 [137]). By incorporating multiple FAD mutations into APP (Swe/Lon/Aus), Koukouli et al. achieved AD-like amyloid deposits, microgliosis, and reactive astrogliosis 12 months after AAV injection into the prefrontal cortex of adult mice, along with NFT pathology [138].

Compared to the dearth of amyloid viral transgenics displaying AD-like pathology, considerably more tau viral transgenic models have been reported. An exhaustive review of viral tau models has recently been published by Cubinkova et al. and we refer readers to this article for more detailed information [139]. Both lentivirus and AAV have been used to express tau in wild-type mice and rats, and both can generate hyperphosphorylated tau in vivo. Both wild-type and mutant (P301L or P301S) tau have been tested, and both can give rise to pathological hyperphosphorylation, although mutant tau produces a more aggressive phenotype [140]. Mature NFTs have been elusive with viral models unless combined with standard APP transgenics [141] (but see [142]), however, a recent study using neonatal intracerebral ventricular (ICV) injection of AAV1-tau-P301L produced widespread tau hyperphosphorylation in wild-type mice followed by thioflavin-S positive NFTs, suggesting that the onset, duration, or multiplicity of transduction may influence the progression of viral tau pathology [143]. Surprisingly, although the mice exhibited behavioral abnormalities, no neuronal loss was observed.

Viral transgenesis has numerous advantages over traditional transgenic models: 1) it is less expensive and faster to generate than germline manipulations; 2) pathology can be regionally targeted, thus allowing analysis of neuronal projections and avoiding peripheral expression; 3) the same virus can often be used in mice or rats, young or old animals, and any region of interest; and 4) viral injection can be combined with genetic models to test functional interactions more quickly and easily than inter-breeding multiple alleles. However, just as with any other system, these benefits come with a cost. First and foremost is mosaicism: not all cells will be transduced, and those that are transduced may take up different numbers of viral particles and express at different levels. This heterogeneity and inter-animal variability necessitates higher animal numbers for each experiment. Second, surgical injection and/or viral transduction may cause an unintended injury response, thus the observed phenotypes may result from an interaction between the expressed viral transgene and the cellular response to injection or transduction. It is therefore important to include a set of animals injected with a control virus encoding a non-pathogenic protein (i.e., GFP or other marker) to confirm that the observed phenotype is transgene-specific. Finally, both AAV and lentivirus are limited in their packaging capacity which prevents the use of large transgenes. For AAV, the maximal transgene size is approximately 4.5 kb, and is not much larger for lentivirus.

### Other tools for specific experimental needs - controllable transgenics

While the first generation transgenic models were useful tools for in vivo studies of protein-protein interactions and cellular responses implicated in AD, their design was rigid with no way to modify where or when the disease-associated protein was produced. Questions that required more selective patterns of transgene expression - for example, expression in mature neurons but not neural progenitors - or which required the ability to turn off expression - for example, mimicking the effect of Aβ-lowering therapies - lead to the creation of new controllable transgenic models based on the tetracycline-transactivator (tTA) expression system. In this bi-partite system, tTA acts as an artificial transcription which binds selectively to an artificial DNA promoter sequence known as the tetracycline response element (*TRE*), both derived from the *E. coli* operon controlling tetracycline resistance. Co-expression of tTA in mice under a selective promoter (the driver line) with a TRE-dependent transgene (the responder line) produces transcription in a pattern dependent on the transgenic tTA promoter. Because the two transgenes are independent of one another, the same responder line can be mated with various driver lines to produce offspring expressing the same transgene in different cell types. When the tTA protein is exposed to tetracycline or its chemical analogs, it undergoes a conformational change that prevents it from binding DNA. Exposure to tetracycline (or the more commonly used doxycycline (dox)) thus provides temporal control over expression of the responder transgene. Two versions of the tTA have been created and are used under different circumstances. The original tet-off system is based on tTA and as its name implies, is active under basal conditions and arrested upon exposure to dox. The subsequent tet-on system based on the reverse tTA (rtTA) is inactive under basal conditions, and opposite from tTA, causes transcriptional activation upon dox administration.

#### tTA-expressing driver lines

Transgene expression with the tet-controlled system requires two parts - a driver line expressing tTA and a responder line expressing the gene of interest under control of a *TRE* promoter - interbred to yield offspring carrying both alleles. Multiple versions of the *TRE* promoter have been created (*tetO, P*_*TRE*_ *, P*_*TRE3G*_, etc.), and all work with the two most common versions of tTA (now known as ‘first’ and ‘second’ generation transactivators, but originally named tTA [144] and tTA-2 [145]). Although these tet-off transactivators are now considered legacy products by their commercial vendor in favor of increasing signal-to-noise improvements in tet-on products, it is the older tet-off system that has been most successful for controllable transgene expression in the mouse brain. Lines expressing tTA or tTA-2 under control of the calcium calmodulin kinase type IIα (*Camk2a* [146]), neurofilament heavy chain (*NEFH* [147]), and kallikrein related-peptidase 8 promoters (*Klk8*; also called Prss19 or neuropsin [30]) have been used to direct controllable transgene expression in neuronal subsets of the entorhinal/limbic areas (*Klk8*), the broader forebrain (*Camk2a*), or throughout the CNS (*NEFH*), as needed for the particular experiment (Table 4). The potential for tTA-controlled transgene expression in other cell types was greatly increased by the introduction of two Cre-dependent tTA transgenic lines from Luo, Roos, and colleagues [148, 149]. These ‘converter’ lines allow tTA-dependent transgene expression in any cell type for which there is a Cre-specific driver, but has the disadvantage of added breeding to combine three independently assorting alleles (Promoter-Cre x Cre-dependent [lox-stop-lox]-tTA x TRE-[gene-of-interest]). The flexibility and strength of this approach was nicely demonstrated by the Allen Institute in recent work showing the expression of TRE-dependent transgenes in a variety of neuronal cell types for which only Cre-expressing driver lines exist [150]. This tripartite system maintained the spatial expression pattern imparted by the Cre driver, while gaining the transcriptional amplification provided by tTA.

**Table 4:** Controllable transgenic lines for APP and Tau

Model	Transgene	Promoter	Availability	Reference
*tTA-Dependent Responder Lines*				
tetO-APP Lines 102 and 107	mo/huAPP_695_ (Swe/Ind)	TetO (first generation TRE from pTet-Splice)	MMRRC 34845-JAX, 34846-JAX	[69]
rTg4510	huMAPT_4R0N_ (P301L)	TRE (first generation TRE from pTRE)	JAX 015815, 024854	[29]
rTg21221	huMAPT_4R0N_ (wt)	TRE (first generation TRE from pTRE)	Karen Ashe	[156]
rTg9191	huAPP_695_ (Swe/Lon)	TRE (first generation TRE from pTRE)	Karen Ashe	[155]
hTau-A152T Line L1	huMAPT_4R1N_ (A152T)	TRE-Tight (second generation TRE)	JAX 028979	[157]
hTau-WT Line L32	huMAPT_4R1N_ (wt)	TRE-Tight (second generation TRE)	JAX 029269	[157]
*tTA-Expressing Driver Lines*				
Camk2a-tTA Line B	tTA (first generation)	mo*Camk2a*	JAX 007004, 003010	[146]
EC-tTA	tTA2 (second generation)	*Nop/Klk8*	MMRRC 031779-MU	[30]
*NEFH*-tTA Line 8	tTA (first generation)	human *NEFH*	JAX 025397	[147]
ROSA:LNL:tTA	optimized/modified tTA (mtTA)	ROSA26-LNL (Cre-dependent)^a^	JAX 011008	[148]
ROSA26-ZtTA	tTA (first generation)	ROSA26-CAG-LβL (Cre-dependent)^b^	JAX 012266 (see also 024107)	[149]

^a^LNL: loxP-(neomycin/poly A)-loxP

^b^LβL: loxP-(β-geo (lacZ-neomycin phosphotransferase fusion)/3x poly A-loxP

Tables 1-–4: Refer to Fig. 1 for illustration of APP mutations.

A number of tet-on (rtTA) driver lines have also been produced but have been generally less successful than tet-off (tTA) lines for CNS expression. Illustrating the difficulty with the tet-on drivers for brain studies, rtTA expression under control of the ubiquitously expressed *ROSA26* promoter or the artificial *CAGGS* promoter produced strong doxycycline-dependent expression of their respective responder transgenes in peripheral tissue, but neither line achieved transgene expression in the brain [151, 152]. The lack of brain expression was attributed to relatively poor CNS permeability of doxycycline required for transcriptional activation with the tet-on system. Indeed, when rtTA has been used for controllable neuronal transgene expression in the brain, doxycycline concentrations up to 30× higher are needed for transgene expression by the tet-on (rtTA) than for transgene suppression by tet-off (tTA) [153]. More recently, a ubiquitously expressed rtTA controlling PGC1α was shown to diminish the aggregation of mutant huntingtin protein in the brain of transgenic mice, however, the effect may have been peripherally mediated [154].

#### TRE-controlled responder lines

A number of tet-controlled responder lines relevant to AD have been created, characterized, and made available through public repositories. Multiple responder lines are available to express APP harboring dual FAD mutations (APP^Swe/Ind^ lines 102 and 107 [69] and APP^Swe/Lon^ line rTg9191 [155]), while tau models are available to express either wild-type (huMAPT_4R0N_ line rTg21221 [156] and huMAPT_4R1N_ line L32 [157]) or FTLD-associated variants (huMAPT^P301L^ line rTg4510 [29] and huMAPT^A152T^ line L1 [157]). Similar to their standard transgenic counterparts, the tet-controlled APP and MAPT strains develop either amyloid plaques or hyper-phosphorylated tau, but can do so on an accelerated timeline compared with traditional models. For example, tetO-APP line 102 used with the Camk2a-tTA driver can develop amyloid plaques by 1–2 months of age (JLJ, unpublished data), while the rTg4510 line (also used with the Camk2a-tTA driver) develops argyrophilic tangles by 4 months and gross forebrain atrophy by 10 months [29]. The aggressiveness of these models is likely due to the high level of transgene expression attained with the tet-transactivator system which can exceed 10× that of endogenous APP or tau, but is by no means the case for every controllable line.

#### Transgene suppression with tet-off models

One of the main reasons for working with the tet-system is the opportunity for temporal control over transgene expression. Doxycycline is commonly used to regulate the system due to better stability than tetracycline; dox has good tissue penetration, is safe for chronic use, and has been well-characterized pharmacologically. Dox can be administered orally via drinking water or chow, with several companies offering standard dox chow formulations in addition to custom compounding. The dose required for transgene suppression via tTA is higher than for veterinary therapeutic use: 50–200 ppm in chow comes to roughly 0.15–0.6 mg per day for an adult mouse, while therapeutic use at 2.5–5 mg/kg PO q12 comes to 0.025–0.05 mg/day [158]. While safe at these doses, doxycycline is not inert: in vitro it can inhibit MMP2 and MMP9 [159, 160] although this effect has not been demonstrated in vivo [161]. More relevant for neurodegeneration studies is the potential anti-inflammatory effect of dox treatment. Where dox has been used as an anti-inflammatory drug, the doses administered are universally higher than used for tTA regulation, in some cases as much as 15× greater [162–165]. The same trend is true in vitro where dox has been found to dampen cytokine responses by cultured microglial cells, but often at concentrations considerably higher than experienced in vivo [164, 166–168]. Minocycline is a stronger anti-inflammatory than doxycycline, and is more commonly used for its anti-inflammatory effect in vivo (for example, see [169–177]). Thus, while there is a potential for inflammatory modulation by dox, it is a considerably weaker agent than minocycline and is usually used in tet-off transgenic models at doses below those shown to suppress microglial function in vivo.

The optimal dose for transgene suppression is influenced by both driver and responder lines and must be determined empirically for each combination. Depending on perdurance of the transgenic protein, maximal suppression is usually attained within days of starting dox treatment [178]. Conversely, the tTA system can be used to activate transgene expression by removing dox from animals reared on the drug [70, 147, 178, 179]. This ‘reverse’ use of dox treatment comes with the risk of diminished transgene expression in animals removed from dox compared with animals never exposed to the drug, especially when treatment is started before transgene expression begins [180] (JLJ unpublished data). Transgene onset after withdrawing dox can be slower than transgene suppression upon dox exposure, and can require up to 2 weeks to reach maximal levels depending on the model and the dosage [181] (JLJ unpublished data). Even under the best conditions, transgene suppression via dox is good but not complete. The TRE controlling the responder transgene contains not only the tet operator sequence that binds tTA but also a minimal CMV promoter to engage transcription. As a result, early versions of TRE-controlled strains produced low levels of transgenic protein even after dox treatment or in the absence of tTA [69, 178, 179]. Later iterations of the *TRE* have shortened the minimal promoter to reduce transactivator-independent expression (i.e., *P*_*TRE3G*_); complementary improvements in tTA-2 pared the transcriptional activation domain to limit activity in the presence of dox [145](http://www.tetsystems.com/science-technology/scientific-figures/). Nevertheless, be aware that transgene suppression is not the same as the absence of expression.

#### The value of tTA controls

Finally, know that tTA and rtTA are themselves artificial transgenes that should be controlled for in your experimental design. Most common (r)tTA lines were produced by random insertion into the genome and may have disrupted one or more genes in the process. In most cases the insertion site has not been identified, and the possibility of bystander hemizygosity at the disrupted locus should always be considered. In addition, tTA expression may have phenotypic effects of its own. Several groups have reported neurodegenerative phenotypes in tTA-expressing transgenic models, including the heavily used Camk2a-tTA line [182, 183]. In each case, dox-rearing abrogated cell loss, suggesting that the active conformation of tTA and not genome disruption was to blame. Peripheral expression of tTA or rtTA has been linked to lung abnormalities, cardiomyopathy, and microphthalmia independent of any responder transgene [184–186](P. Overbeek, unpublished observation). Inclusion of a tTA-only control group can therefore provide valuable reassurance that observed phenotypes are due to the transgenic protein under study and not to artifacts of this powerful but highly artificial expression system.

### Modeling LOAD - incorporating risk alleles for ApoE and TREM2

Recapitulating amyloid or tau pathology in mice requires the use of dominant mutations identified from rare familial cases of early-onset AD or FTLD. This belies the fact that the vast majority of patients develop late-onset AD (LOAD) and do not carry mutations in *APP, PSEN1* or *PSEN2*, and that *MAPT* mutations are not found in AD. Whether these mutation-based, neuropathology-driven models truly capture the key pathological progression of LOAD is an open question. Studies of LOAD have identified multiple genetic factors that influence the risk of AD but which do not directly cause disease. A quarter century ago, Allen Roses and colleagues established the apolipoprotein E ε4 allele (APOEε4) as a major susceptibility factor for LOAD [187]; to date ApoEε4 remains the most common and significant risk factor for LOAD. Recent genome-wide association studies have uncovered >30 additional risk polymorphisms for LOAD [188]. Collectively, these candidate risk genes suggest that astrocytes, microglia, and immune system dysfunction play a significant role in disease pathogenesis [189]. APOE is normally expressed by astrocytes in the CNS, while another strong modifier, the triggering receptor expressed on myeloid cells 2 (TREM2), is expressed exclusively by microglia in the brain [190, 191]. The effects of these polymorphisms are an area of active investigation, and accordingly, multiple mouse lines of APOE and TREM2 have been created. Modeling of other LOAD genes has been more challenging than for *APOE* and *TREM2* due to weak individual effect sizes and the location of risk-associated polymorphisms in non-coding regions of the gene.

#### ApoE

APOE is an apolipoprotein that binds cholesterol to facilitate its transport. The three APOE isoforms in humans differ at residues 112 and 158: ε2 (Cys 112, Cys158), ε3 (Cys112, Arg158) and ε4 (Arg112, Arg158). APOEε4 increases AD risk and ε2 mitigates it. Mouse has only one ApoE isoform and it is believed to resemble human APOEε3 in its biophysical properties [192]. Mice deficient in ApoE were created by gene targeting nearly 25 years ago [193, 194]. Homozygous *ApoE* null mice are viable and overtly healthy but develop hypercholesterolemia and atherosclerotic lesions with age (Table 5). Within the CNS, the *ApoE* null mice display none of the characteristic AD pathologies, but do have impaired adult hippocampal neurogenesis [195], however, the relevance of this phenotype is unclear. The *ApoE* knock-out mice have been crossed with various AD models to test how loss-of-function affects pathology relative to animals expressing one of the three human APOE isoforms (described below).

**Table 5:** APOE and TREM2 Models

Model	Allele	Targeting approach	Availability	Reference
*ApoE Lines*				
GFAP-ApoE3 Line 37, GFAP-ApoE4, Line 1	hu*APOE3* or *E4* cDNA	Transgenic, hu*GFAP* promoter	JAX 004633, 004631	[196]
ApoE2, E3, E4 KI	hu*APOE2, E3,* or *E4*	Targeted insertion of *APOE* cDNAs	NA	[197]
ApoE KO	*Apoe* deletion (exon 3 replacement)	Targeted neo insertion replacing part of *Apoe* exon 3	JAX 002052; 014556	[193, 221]
APOE*3, E*4 KI	hu*APOE3 or E4*	Targeted replacement of *Apoe* exons 2–4	JAX 027894 (E4) JAX 029018 (E3)	NA
APOE2, E3, E4 Targeted replacement	hu*APOE2, E3, or E4*	Targeted replacement of *Apoe* exons 2–4	Taconic	[199–201]
*TREM2 Lines*				
TREM2 KO	*Trem2* deletion (exon 2–3 deletion)	Targeted lacZ/neo replacement of TREM2 exons 2–4	UCD/KOMP VG10093	[206]
TREM2^−/−^	*Trem2* deletion (exon 2–3 deletion)	Targeted deletion TREM2 exons 3–4	Marco Colonna	[205]
TREM2 KO	*Trem2* deletion (Q17X)	CRISPR/Cas9 targeted deletion	JAX 027197	NA
TREM2 R47H KI	endogenous mouse *Trem2* (R47H)	CRISPR/Cas9 targeted mutation	JAX 027918	NA
TREM2 Y38C KI	endogenous mouse *Trem2* (Y38C)	CRISPR/Cas9 targeted mutation	JAX 029725	NA
TREM2 p.T66 M	endogenous mouse *Trem2* (T66 M)	CRISPR/Cas9 targeted mutation	Christian Haass	[208]
TREM2 flox	*Trem2* ^*tm1c(EUCOMM)Wtsi*^ loxP-flanked mouse *Trem2* exons 2–3	Targeted insertion	JAX 029853	NA

Three sets of human APOE mice have been generated. The Holtzman group produced transgenic mice expressing human APOEε3 or APOEε4 cDNA under control of the human GFAP promoter and then removed murine ApoE from the background by crossing the transgenics onto the *ApoE* null line (line 37 for ε3 and line 22 for ε4) [196]. The resulting mice express human APOE in the brain at a level similar to that of adult human cortex. While the mice are useful in examining the role of astrocytic APOE and for comparing the effects of ε3 and ε4, inherent differences in transgene integration site, copy number, and expression level may confound interpretation of differences between the two isoforms. In addition, the need to maintain each transgene on an *ApoE* null background for “humanization” of the model makes further genetic studies complicated.

Bruce Lamb’s group created APOEε2, ε3 and ε4 knock-in mice by targeting human ε2, ε3, and ε4 cDNAs in-frame into the endogenous mouse *ApoE* gene [197]. Because these mice express human APOE under the endogenous mouse promoter, differences between the APOE isoforms can be accurately compared. An added advantage of this strategy is its direct “humanization” by simultaneously inserting the human allele and disrupting mouse *ApoE*. Unfortunately, no isoform-specific differences in brain cholesterol or Aβ levels were detected and the utility of the mice is thus limited.

Through a similar gene-targeting approach, Maeda and colleagues created the so-called APOE targeted-replacement mice in which human *APOEε2* [198], *ε3* [199], or *ε4* [200] alleles were inserted into the endogenous mouse *ApoE* locus (Table 5). Unlike the APOE knock-in series, coding exons 2–4 and the associated intronic sequences of each *APOE* gene were used instead of cDNA and mouse *ApoE* gene was deleted instead of disrupted. These mice exhibit allele-specific differences in both the CNS and periphery and are the most popular APOE mice for testing differential effects of APOE isoforms either on their own or in a model of AD pathology. Although the mice can be purchased through Taconic, there are restrictions on use of the animals. To overcome these limitations, a similar set of APOE-targeted humanization mice was recently created and made available through Jax with few restrictions (APOE*4 KI, Stock No. 027894; APOE*3 KI, Stock No. 029018).

Because the focus of the current review is on practical considerations for best using the models rather than their biological or pathological underpinnings, it will suffice to say that APOE mediates diverse functions in the brain, modifying parenchymal and vascular amyloid pathology, tau-mediated neurodegeneration, and neuroinflammation in an isoform-dependent manner [201–203].

#### TREM2

TREM2 is a type-1 membrane protein expressed in myeloid cells. Autosomal recessive mutations of TREM2 such as Y38C and T66M lead to Nasu-Hakola disease characterized by bone cysts and dementia, believed to arise through loss of function [204]. Rare variants in the TREM2 extracellular domain, particularly R47H, appear to confer LOAD risk in an autosomal dominant manner [190, 191]. The first *Trem2* knock-out strain was created by Colonna and colleagues through targeted deletion of exons 3 and 4 encoding a portion of the transmembrane and cytoplasmic domains [205]. A separate knock-out line was generated by the UC Davis KnockOut Mouse Project (KOMP) through targeted replacement of exons 2, 3, and most of 4 with a lacZ reporter and neomycin resistance cassette [206]. While the KOMP *Trem2* line has proven to be a true loss-of-function allele, the deletion results in upregulation of Treml1 which may complicate interpretation of resulting phenotypes [207]. In contrast, marginal Treml1 upregulation was detected in the Colonna line or in a new knock-out line from Jax made using CRISPR/Cas9 genome editing to delete a 175 base pair fragment and create a stop codon at amino acid 17 (Jax stock #027197). Jax has also created a Cre-conditional allele of *Trem2* by flanking exons 2 and 3 with loxP sites, which will allow peripheral vs. central effects of TREM2 to be dissected (Jax stock #029853). Finally, knock-in lines incorporating the R47H and Y38C (Jax stock #027918 and 029725) or T66M [208] variants have been generated. These mice will be valuable for investigating pathogenic mechanisms of TREM2 in both LOAD and Nasu-Hakola disease.

## Conclusions

Although the neuropathology of AD had long been established, it was not until the discovery of familial mutations responsible for early-onset FAD and FTLD that mouse models capturing the hallmark features of AD became possible. Since the first amyloid model was published in 1995, many dozen more genetically engineered lines have been described with select aspects of AD neuropathology and downstream behavioral and degenerative phenotypes. It is our hope that this review presents an insightful and unbiased coverage of the pros and cons of the various AD mouse models as well as the practical considerations for choosing and using these models. To further facilitate the selection process, below we provide a summary for the most commonly used models (Table 6).

**Table 6:** Quick guide to the pros and cons of commonly used AD mouse models

Model	Main features	Pros	Cons	Examples of use in AD research
Tg2576	mid-life amyloid pathology (10–14 mo)	well-characterized, maintains aging feature of AD	high lethality on C57 background, Tg male aggressive and needs to be single-housed	[222–224]
APP/PS1	early-onset (~6 mo) amyloid pathology	well-characterized, co-integrated transgenes breed as a single allele	like other co-integrated models, cannot control for independent transgene effects	[225–227]
5XFAD	juvenile-onset amyloid pathology (~3 mo)	rapid onset phenotype, co-integrated transgenes breed as a single allele	non-physiological combination of FAD mutations, marked intracellular Aβ accumulation	[228–230]
3xTg-AD	early- to mid-life amyloid pathology plus hyperphosphorylated tau	captures both Aβ and phospho-tau features of AD	variable pathology between colonies and sexes, genetic drift has been observed	[231–233]
rTg4510	early-onset neurofibrillary tangles (~5–6 mo), severe neurodegeneration	temporally controllable, rapid onset phenotype, develops true NFT pathology, well-characterized	breeding complicated by need for two independent transgenes, 13-fold overexpression of tau protein	[67, 234, 235]
PS19	mid-life neurofibrillary tangles (6–9 mo), marked neurodegeneration	single-transgenic model, mid-life onset allows use in experiments expected to either delay or exacerbate pathology	transgene expression in spinal cord causes paralysis by mid-life	[236–238]
APP^NLF^	mid-life amyloid pathology (~12 mo for homozygote, but note >24 mo for heterozygote allele)	endogenous APP level, native human Aβ sequence	limited cognitive impairment, requires homozygous allele for mid-life onset	[59, 239]
APP^NLGF^	juvenile-onset amyloid pathology (~3–4 mo for homozygote, ~9 mo for heterozygote)	endogenous APP level, can be used as heterozygote	non-native Aβ sequence, mild cognitive phenotype	[60, 240]
hTau	mid-life hyperphosphorylated tau (~6 mo)	near-endogenous level expression of all 6 human wild-type tau isoforms	complicated breeding of transgene on null background, mild phenotype variable between colonies	[81, 82]
APOE2, E3, E4 Targeted replacement	allele-specific effects on Aβ, tau, brain atrophy, and neuroinflammation; both central and peripheral functions influenced by allele	widely-studied, expressed at endogenous levels, mouse ApoE deleted	cannot distinguish central vs. peripheral effects; available through Taconic but with restrictions on usage	[201, 241]

Genetics has been and will remain the driving factor in AD mouse model development. While studies of early-onset cases established the importance of neurons in APP/Aβ and tau/NFT pathology, more recent LOAD genes suggest a crucial role for non-neuronal cells, particularly microglia, in disease progression. Future studies would do well to consider changes in the immune system alongside those in neuropathology and cognition. Further, since the vast majority of AD cases arise not from autosomal dominant mutations but through the complex interaction of multiple genetic polymorphisms and environmental risk factors that accrue overtime, future modeling should also shift from single FAD gene targeting to simultaneous manipulation of multiple LOAD genes. With the advent of CRISPR-Cas9 genome editing and with investment from the National Institutes of Health towards resources such as MODEL-AD (https://model-ad.org), we expect major progress in this next phase of AD model development.

## Abbreviations

3R/4R: Three-repeat or four-repeat tau protein
AD: Alzheimer’s disease
ApoE: Apolipoprotein E
APP: Amyloid precursor protein
Aβ: Amyloid β peptide
Arc: Arctic (familial APP mutation)
Aus: Austrian (familial APP mutation)
BACE1: β-APP cleaving enzyme
CAA: Cerebral amyloid angiopathy
CAG: Cytomegalovirus immediate early enhancer combined with the chicken β-actin promoter
Camk2a: Calcium calmodulin kinase type IIα
CBA: Chicken β-actin
CMV: Cytomegalovirus
Dox: Doxycycline
FAD: Familial Alzheimer’s disease
Flo: Florida (familial APP mutation)
FTLD: Frontotemporal lobar degeneration
Ibe: Iberian (familial APP mutation)
Ind: Indiana (familial APP mutation)
Jax: The Jackson Laboratory
KI: Knock-in
Klk8: Kallikrein related-peptidase 8
KOMP: KnockOut Mouse Project
LOAD: Late-onset Alzheimer’s disease
Lon: London (familial APP mutation)
MAPT: Microtubule-associated protein tau
NEFH: Neurofilament heavy chain
NFT: Neurofibrillary tangle
PAC: P1-derived artificial chromosome
PDGFB: Platelet-derived growth factor B chain
Prnp: Prion protein
PSEN1 (or PS1): Presenilin 1
rtTA: Reverse tetracycline transactivator
Swe: Swedish (familial APP mutation)
Thy1: Thymus cell surface antigen 1
TRE: Tetracycline response element
tTA: Tetracycline transactivator
TREM2: Triggering receptor expressed on myeloid cells 2
YAC: Yeast artificial chromosome
